# Analysis of expressed sequence tags and identification of genes encoding cell-wall-degrading enzymes from the fungivorous nematode *Aphelenchus avenae*

**DOI:** 10.1186/1471-2164-10-525

**Published:** 2009-11-16

**Authors:** Nurul Karim, John T Jones, Hiroaki Okada, Taisei Kikuchi

**Affiliations:** 1 Forestry and Forest Products Research Institute, Tsukuba, Ibaraki 305-8687, Japan; 2 Plant-Pathogen Interactions Programme, Scottish Crop Research Institute, Invergowrie, Dundee, DD2 5DA, UK; 3 National Institute for Agro-Environmental Sciences, Tsukuba, Ibaraki, 305-8604, Japan

## Abstract

**Background:**

The fungivorus nematode, *Aphelenchus avenae *is widespread in soil and is found in association with decaying plant material. This nematode is also found in association with plants but its ability to cause plant disease remains largely undetermined. The taxonomic position and intermediate lifestyle of *A. avenae *make it an important model for studying the evolution of plant parasitism within the Nematoda. In addition, the exceptional capacity of this nematode to survive desiccation makes it an important system for study of anhydrobiosis. Expressed sequence tag (EST) analysis may therefore be useful in providing an initial insight into the poorly understood genetic background of *A. avenae*.

**Results:**

We present the generation, analysis and annotation of over 5,000 ESTs from a mixed-stage *A. avenae *cDNA library. Clustering of 5,076 high-quality ESTs resulted in a set of 2,700 non-redundant sequences comprising 695 contigs and 2,005 singletons. Comparative analyses indicated that 1,567 (58.0%) of the cluster sequences had homologues in *Caenorhabditis elegans*, 1,750 (64.8%) in other nematodes, 1,321(48.9%) in organisms other than nematodes, and 862 (31.9%) had no significant match to any sequence in current protein or nucleotide databases. In addition, 1,100 (40.7%) of the sequences were functionally classified using Gene Ontology (GO) hierarchy. Similarity searches of the cluster sequences identified a set of genes with significant homology to genes encoding enzymes that degrade plant or fungal cell walls. The full length sequences of two genes encoding glycosyl hydrolase family 5 (GHF5) cellulases and two pectate lyase genes encoding polysaccharide lyase family 3 (PL3) proteins were identified and characterized.

**Conclusion:**

We have described at least 2,214 putative genes from *A. avenae *and identified a set of genes encoding a range of cell-wall-degrading enzymes. This EST dataset represents a starting point for studies in a number of different fundamental and applied areas. The presence of genes encoding a battery of cell-wall-degrading enzymes in *A. avenae *and their similarities with genes from other plant parasitic nematodes suggest that this nematode can act not only as a fungal feeder but also a plant parasite. Further studies on genes encoding cell-wall-degrading enzymes in *A. avenae *will accelerate our understanding of the complex evolutionary histories of plant parasitism and the use of genes obtained by horizontal gene transfer from prokaryotes.

## Background

The complete genome sequence of the free-living nematode *Caenorhabditis elegans *and the wealth of information on gene expression and function for this nematode [1,2] provide an excellent starting point for genome analysis of other nematodes. For less well studied organisms, where whole genome sequencing is currently unlikely, Expressed Sequence Tag (EST) analysis is a cost-effective method for gene discovery. EST analysis has been widely used within the Phylum Nematoda. However, most effort has been focused on plant or animal parasitic nematodes. Free living nematodes, with the notable exceptions of *C. elegans, C. briggsae *and *Pristionchus pacificus*, remain under represented in terms of ESTs.

*Aphelenchus avenae *is a well-known fungal feeding nematode that is currently placed in the superfamily Aphelenchoidea (family Aphelenchidae) [3]. This nematode is ubiquitous in soil and is associated with saprophytic, pathogenic, and mycorrhizal fungi. As a fungal feeder, *A. avenae *has potential as a bio-control agent against soil-borne fungal plant pathogens [4-8] and, as it has a remarkable ability to survive desiccation; it is also used as a model system for studying anhydrobiosis in animals [9].

Although *A. avenae *is commonly found in soil samples taken from the rhizospheres of diseased and healthy plants, it is widely considered to be incapable of attacking healthy tissues of higher plants [10,11]. It has been suggested that when the nematode is found in association with plant material this occurs as a result of the nematode feeding on fungi associated with the plant. Alternatively, the finding of *A. avenae *within plant tissues [12,13] and its demonstrated ability to reproduce on plant callus material [13,14] may show that it can survive in healthy plant tissues and act as a facultative plant parasite. The role, if any, of *A. avenae *in relation to plant disease therefore remains uncertain.

In a previous study [15], we described the generation, analysis and annotation of over 10,000 ESTs from the pinewood nematode, *Bursaphelenchus xylophilus*, a pathogenic nematode species which was thought to belong to the same superfamily (Aphelenchoidea) as *A. avenae *[3] (but see below) and which can feed on live trees as well as on fungi. Genes encoding a range of cell-wall-degrading enzymes including cellulase (β-1,4-endoglucanase) [16], β-1,3-endoglucanase[17], pectate lyase [18] and expansin [19] were subsequently identified and characterized from this nematode. Similar enzymes [20-33] have also been identified and characterized from other plant parasitic nematodes including cyst and root-knot nematodes. These enzymes are produced within the esophageal gland cells of the nematode, secreted through the nematode stylet into host tissues and are thought to play an important role in the host-parasite interaction, allowing invasion and migration of the nematode through plant tissues. The presence of these enzymes is unusual; they are not usually present in animals and it is thought that the genes encoding them may have been acquired by horizontal gene transfer [34,35].

**Figure 1:**
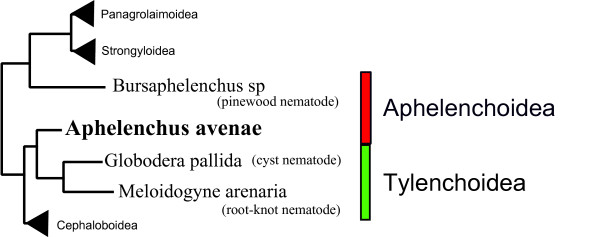
**Simplified tree showing relationships of *Aphelenchus avenae*, *Bursaphelenchus *and cyst/root-knot nematodes**. Recently published phylogenetic tree based on SSU of ribosomal DNA [38] has been adapted for drawing this simplified tree. Taxonomic positions are indicated based on superfamily [3].

In classical taxonomic classification, *A. avenae *(family Aphelenchidae) has been placed in the same superfamily (Aphelenchoidea) as *B. xylophilus *(family Aphelenchoididae) whereas cyst and root-knot nematodes are placed in a different superfamily, the Tylenchoida (family Tylenchida), although the three nematode groups are all placed within the infraorder Tylenchromorpha [3]. However, recent phylogenetic studies using ribosomal DNA suggest that *A. avenae *is more closely related to cyst and root-knot nematodes than it is to *B. xylophilus *[36-38]. The current view of the taxonomy of three nematode groups is summarized in Fig. 1.

Although some of the parasitism genes are common to both superfamiles, Aphelenchoidea (*Bursaphelenchus*) and Tylenchoidea (cyst and root-knot nematodes), there are also differences between the parasitism genes present in the two nematode groups [3]. For example, *Bursaphelenchus *and cyst/root-knot nematodes contain endogenous expansins and pectate lyases which appear to have been acquired by a common ancestor via horizontal gene transfer [18,19]. However, the cellulases in the two groups are different. Those present in cyst and root-knot nematodes are from glycosyl hydrolase family 5(GHF5) and are likely to have been acquired from bacteria whereas those in *Bursaphelenchus *are from GHF45 and appear to have been acquired from fungi [35]. Nothing is currently known about such pathogenicity genes in *A. avenae *and the presence or absence of such genes in this nematode may shed light onto whether this nematode can act as a plant parasite. In addition, the presence of such horizontally acquired genes in *A. avenae *may also help reveal the evolutionary history of these genes within nematodes.

To address these issues, we have generated over 5,000 high quality ESTs from a mixed-stage *A. avenae *cDNA library. We report the identification of genes that could encode enzymes that degrade the cell walls of plants or fungi. We have also analysed the clustered *A. avenae *sequences using the Gene Ontology (GO) classification system and undertaken comparative analysis with *C. elegans *and other nematode protein databases.

## Results and Discussion

### Generation of ESTs from an *A. avenae *cDNA library

A mixed-stage *A. avenae *cDNA library (Aamk) was constructed to generate ESTs (Table 1). Sixteen clones were randomly selected and the sizes of the inserts in these clones were assessed after digestion with appropriate restriction enzymes. These insert sizes ranged from 400 to 1,600 base pairs (bp) with an average of 1.1 kilobase pairs (kb). A total of 5,472 cDNA clones were subsequently randomly isolated and sequenced from the 5' end in order to generate ESTs. The sequences were trimmed of vector sequence, adaptor sequence, poly(A) tail and low-quality sequence and filtered for minimum length (150 bp), resulting in a total of 5,076 high quality ESTs. The average length of submitted ESTs was 468 nucleotides (nt).

**Table 1:** *A. avenae *cDNA library, ESTs and clusters statistics

Titre of cDNA library (pfu/ml)	1.2 × 10^5^
Average cDNA insert size	1.1 kb
Total cDNA clones picked and sequenced	5472
Sequences passing quality check	5076 (93%)
Average length	468 bp
Singletons	2005
Contigs	695
Total number of clusters	2700

### Cluster formation and analysis

**Figure 2:**
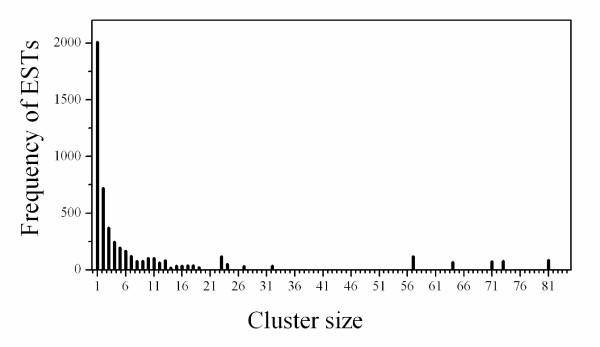
**Histogram showing the distribution of ESTs from *A. avenae *by cluster size**. For example, there were five clusters of size 23 containing a sum total of 115 ESTs. Distribution of contig sizes is not shown.

To identify overlapping EST sequences, improve base accuracy and transcript length, and to produce non-redundant EST data for further functional annotation and comparative analysis, the 5,076 ESTs from the *A. avenae *library were grouped by sequence identity into clusters. Based upon regions of nucleotide identity, EST sequences were merged into contiguous consensus sequences (contigs). 'Contig' member ESTs derive from identical transcripts while 'cluster' members may derive from the same gene but represent different transcript splice isoforms (*i.e *ESTs form contigs, contigs form clusters). Two thousand seven hundred non-redundant EST clusters were generated from the ESTs (Table 1). In 2,005 cases, clusters consist of a single EST, whereas the largest single cluster contains 81 ESTs (1 case) (Fig. 2). The majority of contigs (650 out of 695) were composed of 2-10 ESTs. 89 clusters were found to contain multiple contig members, revealing potential splice isoforms. By eliminating redundancy during this contig building, the total number of nucleotides used for further analysis was reduced from 2.82 million to 1.61 million. In addition, this process significantly increased the length of assembled transcript sequences from 468 ± 114 nt for submitted ESTs alone to 595 ± 321 nt for contigs. The longest sequence generated also increased from 724 to 2,154 nt.

Based on the identified clusters 2,700 *A. avenae *genes were identified, corresponding to a new gene discovery rate of 53% (2,700/5,076). However, 2700 clusters is likely to be an overestimate of the true gene discovery rate, as one gene could be represented by multiple nonoverlapping clusters. Such "fragmentation" has been estimated at 18% using *C. elegans *as a reference genome [39]. After allowing for such potential fragmentation, we estimated that the *A. avenae *sequences derived from minimum of 2,214 genes giving a discovery rate of 44% (2,214/5,076). Assuming between 14,000 and 21,000 total genes, the range encompassed by *Meloidogyne hapla *[40], *M. incognita *[41] and *C. elegans *(Wormpep v. 203), the cluster dataset could represent approximately 11-16% of *A. avenae *genes.

### Transcript abundance and highly represented genes

A high level of representation in a cDNA library usually correlates with high transcript abundance in the original biological sample [42], although artifacts of library construction can result in a selection for or against some transcripts. The *A. avenae *clusters were ranked according to the number of contributing ESTs, and the top 25 clusters are summarized in Table 2. Each of these clusters contained fifteen or more EST copies and represented 16% of the total number of ESTs obtained. Eighteen of the clusters had significant matches to genes with annotated functions based on BLASTX (E < 1e-5) against the non-redundant database, and all of these had homologues in nematodes. Transcripts abundantly represented in the *A. avenae *library included genes encoding structural proteins (such as actin, collagen, tropomyosin and troponin C) and proteins which carry out core metabolic processes (*e.g*. cytochrome c oxidase, ATP synthase). Other abundant ESTs included a small heat shock protein and phosphoenolpyruvate carboxykinase. The latter enzyme has previously been cloned from the parasitic nematodes, *Haemonchus contortus *and *Ascaris suum *[43,44]. Cluster AAC00541, containing 23 ESTs, was similar to an SXP/RAL-2 family protein from the parasitic nematode, *Anisakis simplex*. Similar genes have previously been characterized from plant parasitic nematodes [45], and individual genes have been shown to be expressed in a range of secretory tissues including the gland cells surrounding the main sense organs (amphids) and the hypodermis.

**Table 2:** The most abundantly represented transcripts in the *A avenae *cDNA library

			Non-redundant GenBank			
			
No.	Cluster ID	ESTs	Best identity descriptor	Accession	E-value	*C. elegans *gene
1	AAC00129	81	Actin [*Panagrellus redivivus*]	AAM47606	0.0	M03F4.2
2	AAC00161	73	Hypothetical protein F42A10.7 [*C. elegans*]	NP_498341	4e-40	F42A10.7^a^
3	AAC00061	71	Small heat shock protein OV25-2 [*Onchocerca volvulus*]	P29779	5e-10	F08H9.4
4	AAC00128	64	Collagen family member (col-144) [*C. elegans*]	NP_505374	1e-25	B0222.6^a^
5	AAC00023	57	Troponin C [*C. brenneri*]	ACD88888	1e-71	F54C1.7
6	AAC00091	57	Tropomyosin [*Heligmosomoides polygyrus*]	ABV44405	2e-82	Y105E8B.1^a^
7	AAC00187	32	Cuticular collagen [*Teladorsagia circumcincta*]	CAA65506	3e-30	T07H6.3
8	AAC00105	27	Hypothetical protein CBG24046 [*C. briggsae*]	CAE56371	2e-66	F09F7.2^a^
9	AAC00123	24	Novel	--	--	--
10	AAC00255	24	Novel	--	--	--
11	AAC00048	23	Temporarily assigned gene name family member [*C. elegans*]	NP_501440	1e-141	T01B11.4
12	AAC00088	23	Novel	--	--	--
13	AAC00109	23	Hypothetical protein CBG15446 [*C. briggsae*]	XP_001677444	3e-43	ZK721.2
14	AAC00321	23	Novel	--	--	--
15	AAC00541	23	SXP/RAL-2 family protein [*Anisakis simplex*]	BAF43534	2e-14	ZK970.7
16	AAC00269	19	Hypothetical protein CBG02561 [*C. briggsae*]	XP_001679653	3e-08	ZK84.1^a^
17	AAC00121	18	Cytochrome c oxidase subunit I [*Steinernema carpocapsae*]	YP_026087	1e-153	MTCE.26
18	AAC00143	18	Cuticle preprocollagen [*Meloidogyne incognita*]	AAC48358	9e-22	F41F3.4
19	AAC00163	17	Phosphoenolpyruvate carboxykinase [*Haemonchus contortus*]	P29190	1e-151	R11A5.4^a^
20	AAC00313	17	ATP synthase subunit family member (atp-2) [*C. elegans*]	NP_498111	1e-149	C34E10.6^a^
21	AAC00112	16	Cytochrome c oxidase subunit III [*Ascaris suum*]	NP_006947	7e-70	MTCE.23
22	AAC00295	16	Novel	--	--	--
23	AAC00024	15	Hypothetical protein CBG21920 [*C. briggsae*]	XP_001678358	2e-21	R06C7.4^a^
24	AAC00265	15	Novel	--	--	--
25	AAC00148	14	Novel	--	--	--

^a ^*C. elegans *homolog has higher probability match than the best GenBank descriptor

Seven of the 25 most abundantly represented transcripts from *A. avenae *had no significant similarity to any sequence in the non-redundant protein database (Table 2). Since most nematode data are available only as ESTs and therefore not included in the BLASTX analysis, we compared these 7 contigs against dbEST using BLASTN and TBLASTX. However, these searches returned no significant matches (E < 1e-5). We also conducted BLASTN and TBLASTX searches against the non-redundant nucleotide database for these sequences. Six of the clusters did not return any matches from this database but cluster AAC00148 produced a match using TBLASTX analysis (E < 1e-5) (Table 2).

### Comparisons to proteins from other species

**Figure 3:**
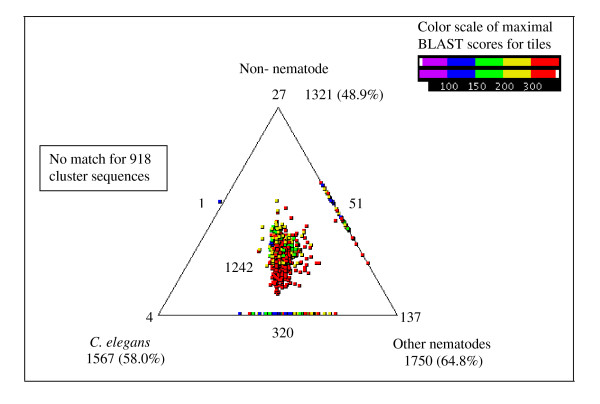
**Comparison of *A. avenae *cluster sequences with *C. elegans*, other nematodes and non-nematode protein sequence databases using SimiTri**. The numbers at each vertex indicate the number of cluster sequences matching only that specific database. The numbers on the edges indicate the number of cluster sequences matching the two databases linked by that edge. The number within the triangle indicates the number of *A. avenae *genes with matches to sequences in all three databases.

We compared the 2,700 cluster sequences from *A. avenae *against three databases containing protein sequences from different organisms. The cluster sequences were compared with protein sequences from (i) *C. elegans *(WORMPEP v.203 [46]), (ii) other nematodes (available protein sequences and peptides from conceptually translated ESTs), and (iii) organisms other than nematodes (from the NCBI non-redundant protein database) [47]. 66% of the *A. avenae *clusters (1,782 of 2,700) had matches in one or more of the three databases and these matches were represented using SimiTri [48] (Fig. 3). In the majority of cases where homologies were found (1,242/1,782), matches were found in all three databases surveyed. Gene products in this category are generally widely conserved across metazoans and many are involved in core biological processes. Examination of the individual database searches showed that 1,567 (58.0%) had homologues in *C. elegans*, 1,750 (64.8%) in other nematodes and 1,321 (48.9%) in organisms other than nematodes. The 918 clusters (34.0%) which had no significant similarity to any sequences in these three protein databases were searched against non-redundant nucleotide and dbEST databases using BLASTN and TBLASTX (employing a cut-off of 1e-05). 56 clusters generated matches in these searches but no matches were obtained for the remaining 862 sequences (31.9%).

Table 3 shows the 15 gene products with the highest level of conservation (E-value ranging from 0 to e-151) between *A. avenae *and *C. elegans*; these include gene products involved in cell structure (for example, actin, UNC-87), protein biosynthesis or regulation (for example, UBQ-1, elongation factor,) and metabolism (for example, enolase, cytochrome c oxidase). Representation of these clusters in the *A. avenae *EST collection varied from 81 ESTs to 2 ESTs. None of these most conserved gene products were nematode specific. Out of all clusters, 461 (17.1%) had homology only to nematodes, either *C. elegans *(4), other nematodes (137), or both (320) (Fig. 3). The most conserved (1e-111) of these nematode-specific proteins was a homolog of a serine proteinase inhibitor, previously characterized from *C. elegans *(K10D3.4) and parasitic nematodes [49,50]. Among the other most conserved nematode-specific clusters were homologs of previously characterized *C. elegans *structural proteins (for example cluster, AAC01973 matched to a collagen family protein, COL-176) as well as uncharacterized *C. elegans *hypothetical proteins (for example, cluster, AAC01948 matched *C. elegans *gene, C34E7.4 which has no known function).

**Table 3:** Most conserved nematode genes between *A. avenae *and *C. elegans*

*A. avenae *cluster	ESTs	Wormpep Accession	*C. elegans *Gene	Assignment	E-value
AAC01067	2	CE01921	F25B5.4	UBQ-1, ubiquitin like protein	0
AAC00578	8	CE18826	H28O16.1	ATP synthase alpha and beta subunits	0
AAC00675	6	CE36954	T21B10.2	ENOL-1, enolase	0
AAC00196	10	CE33098	F46H5.3	Kinase, protein-id: AAB37022.2	0
AAC00129	81	CE12358	MO3F4.2	ACT-4, actin 4	0
AAC00222	7	CE01270	R03G5.1	ELF-4, elongation factor 1-alpha	0
AAC00358	2	CE31915	F25B5.4	UBQ-1, ubiquitin like protein	1e - 178
AAC00334	11	CE36924	F08B6.4	UNC-87, calponin	1e - 177
AAC00339	4	CE02477	C14F11.1	Aspartate aminotransferase	1e - 166
AAC00183	3	CE07458	T01C8.1	AAK2, protein kinase	1e - 160
AAC01744	3	CE18971	T27E9.7	ABCF-2, ABC transporter	1e - 160
AAC00121	18	CE35350	MTCE.26	Cytochrome c oxidase subunit 1	1e - 153
AAC00163	17	CE36360	R11A5.4	Protein-id:CAF31483.1	1e - 152
AAC00313	17	CE29950	C34E10.6	ATP-2, ATP synthase beta subunit	1e - 151
AAC00262	8	CE39807	Y105C5B.28	GLN-3, glutamine synthetase	1e - 151

The 137 cluster sequences where homologs were present only in other nematodes were further categorized based on their BLAST (BLASTX and TBLASTX) results (Additional file 1). Matches were found in plant parasitic, animal parasitic and free living nematodes. 24.8% of sequences (34 of 137) had homology only to sequences from plant parasitic nematodes. Some of these sequences were similar to previously characterized cell-wall-degrading enzymes, which are known to be involved in the parasitism process of these nematodes. For example, cluster, AAC01592 matched an expansin-like protein from *B. xylophilus *[19] and cluster, AAC02968 matched a β-1,4-endoglucanase precursor from *Globodera rostochiensis *[20]. Further analysis of some of the cell-wall-degrading enzymes present in *A. avenae *is presented below.

### Identification of transcripts similar to stress-response genes related to desiccation

BLASTX (E < 1e-5) searches of *A. avenae *cluster sequences against nr protein databases allowed identification of genes that can encode proteins or enzymes important in providing protection against desiccation or other environmental-stress (Additional file 2). One notable observation was the presence of sequences similar to late embryonic abundant (LEA) proteins, which are known to be associated with tolerance to water stress resulting from desiccation [9]. Protein aggregation during desiccation is likely to be a major potential hazard for anhydrobiotes; LEA proteins may act as molecular chaperones or molecular shields and play an important role in the prevention of this aggregation [51]. Thirteen ESTs, distributed in three clusters, (AAC00729, AAC00888, and AAC01781) were identified as having significant similarity to LEA proteins. Cluster, AAC01781 which was identified as a singleton matched a previously characterized LEA protein from desiccated *A. avenae *[9]. In addition, we also identified multiple copies of cytochrome P450, superoxide dismutase, glutathione peroxidase, and glutathione S-transferase, enzymes involved in protection against oxidative damage. Desiccation stress of nematodes caused significant up-regulation of transcripts encoding these genes [52,53].

### Functional classification based on gene ontology

**Figure 4:**
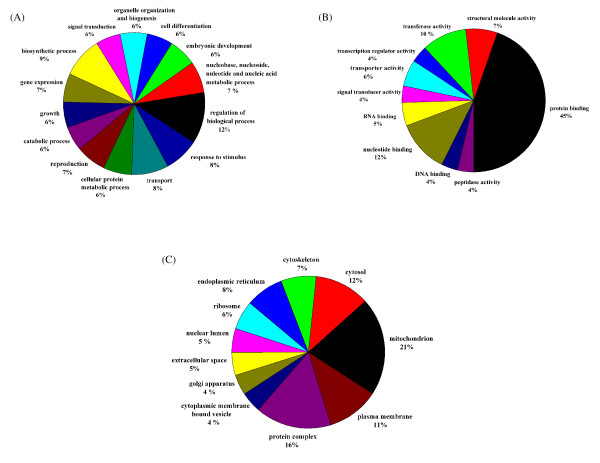
**Summary of the Gene Ontology annotation as assigned by BLAST2GO**. (A) Most represented GO terms (based on number of represented sequences) of the main category "biological process"; (B) Most represented GO terms of the main category "molecular function (C) Most represented GO terms of the main category "cellular component". Multi-level pie charts were generated using the sequence cut-offs 140, 50 and 40 for "biological process", "molecular function" and "cellular component", respectively.

Gene Ontology (GO) has been used widely to predict gene function and classification [54]. BLAST2GO [55,56], a universal, web-based annotation tool was used to assign GO terms for the *A. avenae *cluster sequences, extracting them from each BLAST hit against Swiss-Prot obtained by mapping to extant annotation associations. 1222 sequences out of 2,700 did not retrieve any BLAST results within the set E-value threshold (< 1e-5). Mapping of GO terms and annotation were not possible for 173 and 205 sequences, respectively. The remaining 1,100 (40.7%) sequences were successfully annotated and mapped to one or more of the three organizing principles of GO: biological process, molecular function and cellular component. The matches obtained from this analysis are summarized in Figs 4A-4C.

1,003 of the *A. avenae *cluster sequences generated matches in the "molecular function" class, 933 in the "biological process" class and 924 in the "cellular component" class. Within the "biological process" class the "regulation of biological process (GO:0050789)", "biosynthetic process (GO:0009058)" and "transport (GO:0006810)" categories were the most represented followed by "response to stimulus (GO:0050896) ", based on the annotation assigned by BLAST2GO (Fig. 4A). Within the "molecular function" class the "protein binding (GO:0005515)" term is the most represented followed by "nucleotide binding (GO:0000166)" and "transferase activity (GO:0016740)" (Fig. 4B). Many cluster sequences encoding ribosomal proteins as well as highly expressed genes coding for structural molecules (such as actin) and regulatory molecules (such as transcription factors) are assigned to the "protein binding" term. Since those clusters are abundantly present in the dataset, this may cause overrepresentation of the "protein binding" term. Within the "cellular component" class the "mitochondrion (GO:0005739)" is the most highly represented (Fig. 4C). A complete listing of GO mappings assigned for the *A. avenae *cluster sequences is provided in Additional file 3.

### Identification of transcripts encoding cell-wall-degrading enzymes

BLASTX analysis allowed us to identify various genes with significant similarity to genes encoding enzymes which degrade plant and fungal cell walls (Table 4). The plant cell-wall-degrading enzymes that were identified included cellulase, pectate lyase, polygalacturonase and expansin, while transcripts encoding fungal cell-wall-degrading enzymes included β-1,3-endoglucanase and chitinase.

**Table 4:** *A. avenae *transcripts similar to plant or fungal cell-wall-degrading enzymes

Enzyme name	Cluster ID	ESTs	Non-redundant GenBank		
			
			Best identity descriptor	Accession	E-value
Cellulase	AAC00199	2	β-1,4-endoglucanase [*Pratylenchus penetrans*]	BAB68522	1e-26
	AAC00418	1	β-1,4-endoglucanase [*P. penetrans*]	BAB68522	1e-22
	AAC00801	2	β-1,4-endoglucanase precursor [*Globodera rostochiensis*]	AAC63989	5e-49
	AAC00947	1	β-1,4-endoglucanase [*P. penetrans*]	BAB68522	6e-15
	AAC01152	6	β-1,4-endoglucanase-1 precursor [*Radopholus similis*]	ABV54446	1e-79
	AAC02961	1	β-1,4-endoglucanase-1 precursor [*Heterodera glycines*]	AAC48327	2e-45
	AAC02968	1	β-1,4-endoglucanase precursor [*G. rostochiensis*]	AAC48325	3e-11
	AAC03021	1	β-1,4-endoglucanase precursor [*G. rostochiensis*]	AAD56392	2e-08
					
Pectate lyase	AAC01467	1	Pectate lyase [*Bursaphelenchus mucronatus*]	BAE48373	3e-38
	AAC01649	1	Pectate lyase [*B. mucronatus*]	BAE48373	1e-59
	AAC02466	1	Putative secreted lyase [*Streptomyces ambofaciens*]	CAJ90085	6e-25
	AAC02527	1	Pectate lyase [*B. mucronatus*]	BAE48373	4e-13
	AAC02949	1	Pectate lyase [*B. mucronatus*]	BAE48373	6e-42
	AAC03048	1	Pectate lyase [*B. mucronatus*]	BAE48373	4e-43
					
Polygalacturonase	AAC02157	1	Extracellular polygalacturonase [*Aspergillus clavatus*]	XP_001272239	3e-44
	AAC02706	2	Polygalacturonase precursor [*A. parasiticus*]	P49575	1e-37
					
Expansin	AAC01185	2	Expansin-like protein [*B. xylophilus*]	BAG16537	5e-42
	AAC01434	1	Expansin-like protein [*B. xylophilus*]	BAG16537	5e-25
	AAC01592	1	Expansin-like protein [*B. xylophilus*]	BAG16537	8e-25
	AAC02804	1	Expansin-like protein [*B. xylophilus*]	BAG16537	5e-51
					
1,3-Glucanase	AAC00494	2	Glcosyl hydrolase 16 precursor [*Pedobacter sp*.]	ZP_02027413	8e-42
					
Chitinase	AAC00552	2	Conserved hypothetical protein [*Culex pipiens quinquefasciatus]*	XP_001849531	7e-09
	AAC01706	2	Chitinase family member [*C. elegans*]	NP_508588	3e-45
	AAC02660	1	Putative chitinase [*Ascaris suum*]	AAK93964	4e-75

Eight cellulase genes were present in 8 different *A. avenae *clusters and in all cases homologues were found in other plant parasitic nematodes. One cellulase gene (AAC01152) was identified as a contig of six individual ESTs. Two clusters (AAC00199 and AAC00801) contained 2 ESTs each and remaining cellulase clusters were present as a singleton. Two types of pectin degrading enzymes: pectate lyase and polygalacturonase were identified (Table 4). While all transcripts encoding pectate lyase genes were identified as singletons, polygalacturonase clusters contained either single or two individual ESTs. The features of the sequences of cellulase and pectate lyase are discussed in more detail below.

In addition to the plant cell-wall-degrading enzymes, we identified genes encoding expansin-like proteins in the *A. avenae *dataset. Expansins and expansin-like proteins have recently been described in several plant parasitic nematodes [19,31-33] and it is thought that these proteins disrupt non-covalent bonds in the plant cell wall, enhancing the activity of other enzymes such as cellulases. All four expansin-like transcripts were identified as a best match with the expansin genes from the *B. xylophilus *[19].

Two different types of genes, β-1,3-endoglucanase and chitinase, that could encode enzymes, important in degradation of the fungal cell wall were identified. A gene encoding a β-1,3-endoglucanase has been cloned and characterized from *B. xylophilus *[17] and is thought to aid fungal feeding in this nematode. Chitinase, an enzyme responsible for breakdown of β-1,4-glycosidic bonds within chitin, has been found in wide ranges of nematodes. Since chitin is known to be present in the eggshell [57] and the microfilarial sheath [58] of nematodes, it has been suggested that chitinases have a role in remodeling processes during the molting of filariae and in the hatching of larvae from the eggshell [59,60]. However, the existence of large families of chitinases in the free-living nematode *C. elegans *suggests that these enzymes may also fulfill other functions [61]. In the plant parasitic nematode, *Heterodera glycines*, chitinase was found to be expressed in the subventral oesophageal gland cells of the parasitic stages of this nematode, suggesting a role in parasitism but not in hatching [62]. The fungal feeding plant parasitic *B. xylophilus *also contains chitinase [15]. Since β-1,3-glucan and chitin are the two major structural polysaccharides of the fungal cell-wall, it is possible that fungal feeding nematodes like *B. xylophilus *and *A. avenae *secrete these enzymes in order to metabolize or soften the fungal cell wall as part of the feeding process.

### Characterisation of genes encoding cellulases and pectate lyases; analysis of sequences, phylogentics and expression analysis

Cellulase and pectate lyase have been identified and characterized in a wide range of plant parasitic nematodes [16,18,20-29]. The presence of genes encoding cell-wall-degrading enzymes in *A. avenae *opens up a new avenue for further molecular studies aimed at understanding their functional role in this nematode and investigating the origin and evolution of these genes within the Nematoda. We therefore cloned the full length cDNA and genomic sequences of two putative cellulases (named *Aa-eng-1 *and *Aa-eng-2*) and two putative pectate lyases (named *Aa-pel-1 *and *Aa-pel-2*) and compared these sequences to those from other plant parasitic nematodes.

The full-length sequences of the cellulases were identified from two plasmid clones whose EST sequences are part of cluster, AAC001152 (Table 4). Although, six individual ESTs, form a contig to represent this cluster, one EST was selected as it showed a slightly different nucleotide sequence from the other five ESTs. Two different plasmid clones containing the full length cDNA sequences of the cellulases were subsequently selected and sequenced using the specific primers listed in Table 5.

**Table 5:** Primers used in the analysis of cell-wall-degrading enzymes

Primers	Sequence (5' to 3')	Use
Eng1-0F	CTCTACGGGATGAAGTGTCT	*In situ *hybridization, amplification of cDNA and gDNA of *Aa-eng-1 *and *Aa-eng-2*
Eng1-0R	TTAACAAAAGCGGTACAAG	*In situ *hybridization, amplification of cDNA and gDNA of *Aa-eng-1 *and *Aa-eng-2*
Eng1-1F	GCTCAAGGTCGTCGTCGAGG	Sequencing of cDNA and gDNA of *Aa-eng-1 *and *Aa-eng-2*
Eng1-10F	GGCATCTCCGAGGCCGACG	Sequencing of gDNA of *Aa-eng-1 *and *Aa-eng-2*
Eng1-10R	CTTGCCGTACTCCTGCGCGAT	Sequencing of gDNA of *Aa-eng-1 *and *Aa-eng-2*
Eng2-20R	GCTACTTTGCTGGTCCACGT	Sequencing of gDNA of *Aa-eng-2*
Pel1-0F	TCCGACGACAACGTCAACCA	*In situ *hybridization, amplification of cDNA and gDNA of *Aa-Pel-1*
Pe11-0R	AAACCCTCAGCATGTTTGATAC	*In situ *hybridization, amplification of cDNA and gDNA of *Aa-Pel-1*
Pel1-1F	TCGAGAACGTCTGGTGGGA	Sequencing of cDNA and gDNA of *Aa-Pel-1 *and *Aa-Pel-2*
Pe11-10F	CTTGGAGGTACGCTTCGTACG	Sequencing of gDNA of *Aa-Pel-1*
Pe11-10R	TGACCTTCTTCGCCGCAGTG	Sequencing of gDNA of *Aa-Pel-1*
Pe11-20F	GAAATGGTACGATTAGTCCTG	Sequencing of gDNA of *Aa-Pel-1*
Pe12-0F	TCAGTCGGACAGCTTTTCCTC	Amplification of cDNA and gDNA of *Aa-Pel-2*
Pe12-0R	AGCAGGCATTTCGTCGACAC	Amplification of cDNA and gDNA of *Aa-Pel-2*
Pe12-10F	CGAGATGGCACGGGTGCCGA	Sequencing of gDNA of *Aa-Pel-2*
Pe12-10R	CGTAGCGAGAAATTTTCGATCA	Sequencing of gDNA of *Aa-Pel-2*

**Figure 5:**
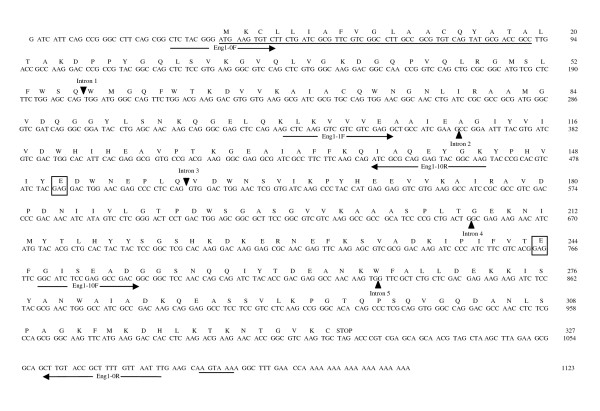
***Aa-eng-1*cDNA sequence and predicted amino acid sequences**. The predicted signal peptide for secretion and polyadenylation signal sequence are underlined. Predicted positions of the five intron sequences identified are indicated by darkened triangles. Primers used for obtaining the full length cDNA sequence and genomic amplification are indicated by arrows. The amino acids within the boxes represent the predicted active site residues.

The *Aa-eng-1 *cDNA was 1,104 bp in length (excluding the polyA tail) and included a 981-bp open reading frame (ORF) that could encode a protein of 327 amino acids with an ATG start codon at position 35 and a TGA stop codon at position 1,016 (Fig. 5). The complete cDNA of *Aa-eng-2 *was 1,107 bp in length and also contained a potential ORF of 327 amino acids with an ATG start codon at position 35 and TGA stop codon at 1,016. A signal peptide of 19 amino acids is predicted by SignalP [63] at the N terminus of the deduced AA-ENG-1 and AA-ENG-2 polypeptides. The predicted molecular masses of the putative mature proteins were 34.130 kDa and 34.059 kDa respectively and the theoretical pI value was 6.2 for both proteins. The AA-ENG-1 and AA-ENG-2 proteins contained a catalytic domain homologous to GHF5 β-1,4-endoglucanases as predicted by PRODOM [64]. The deduced amino acid sequences showed highest similarity with the GHF5 endoglucanase from the migratory plant parasitic nematode *Radopholus similis *(GenBank Accession No. [ABV54446]). The highest non-nematode similarities of both AA-ENG-1 and AA-ENG-2 were with the β-1,4-endoglucanase from *Cytophaga hutchinsoni *(cellulolytic gliding bacterium; GenBank Accession No. [YP_678708]). AA-ENG-1 and AA-ENG-2 share 99% identity in their amino acid sequences.

Genomic clones of *Aa-eng-1 *and *Aa-eng-2 *were obtained by PCR amplification using gene-specific primers (Table 5) and genomic DNA as template. The *Aa-eng-1 *and *Aa-eng-2 *genomic DNA products were1,518 bp and 1,522 bp long respectively from the ATG to the stop codon. The position of exon/intron boundaries of the genomic sequences were determined by aligning the genomic sequences with the corresponding cDNA sequences. All introns were bordered by canonical cis-splicing sequences [65]. Five introns were identified in *Aa-eng-1 *(Fig. 5) of which four introns were small (40 bp to 85 bp) a feature commonly found in nematodes [66]. Only the first intron was larger (319 bp). Five introns were also identified in *Aa-eng-2*. The first intron was 337 bp long and the length of remaining four introns ranged from 40 to 72 bp. The intron positions of *Aa-eng-1 *and *Aa-eng-2 *genes were identical to each other.

Sequence alignment of the two endoglucanases from *A. avenae *with GHF5 endoglucanases from nematodes and other organisms revealed that both AA-ENG-1 and AA-ENG-2 possess a consensus pattern of GHF5 endoglucanases in their primary amino acid sequences in which two glutamic acids residues are the predicted proton donor and mucleophile/base of the catalytic site (Fig. 5) which is also true for all previously described nematode GHF5 endoglucanases. In addition to the catalytic domain some of these proteins contain a cellulose binding domain (CBD) joined to the catalytic domain through a linker peptide. The GHF5 endoglucanase genes isolated from plant parasitic nematodes have also different structures: all have a signal peptide and catalytic domain, some have an additional linker and CBD and others only have a linker but no CBD [25,67]. However, neither peptide linkers nor CBD domains were present in the two GHF5 endoglucanases isolated from *A. avenae*. Expansins from cyst nematodes have also been shown to contain a CBD, but no such domains were predicted in other sequences within the EST dataset, including the putative expansins.

**Figure 6:**
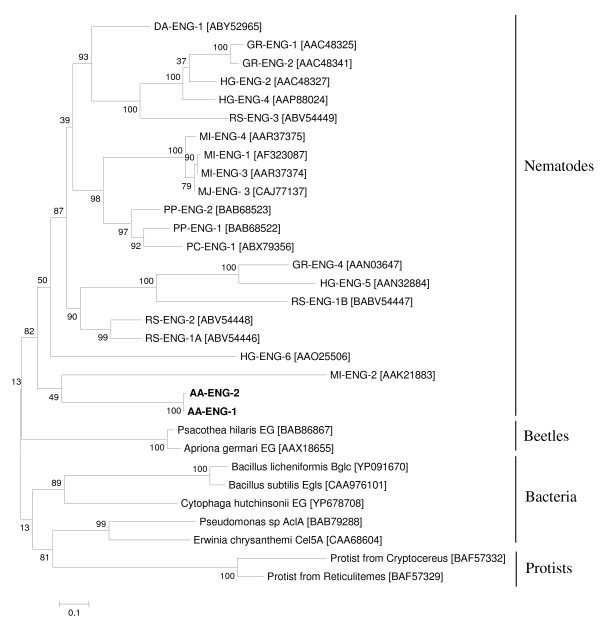
**Unrooted phylogenetic tree of GHF5 catalytic domains based on the protein sequences using the maximum likelihood method**. The GHF5 proteins from *A. avenae *(AA-ENG-1 and AA-ENG-2) are labeled in bold. GenBank accession numbers of GHF5 proteins from *Meloidogyne incognita *(MI-ENG-1, MI-ENG-2, MI-ENG-3 and MI-ENG-4), *Pratylenchus penetrans *(PP-ENG-1 and PP-ENG-2), *Pratylenchus coffeae *(PC-ENG-1), *Radopholus similis *(RS-ENG-1A, RS-ENG-1B, RS-ENG-2 and RS-ENG-3), *Globodera rostochiensis *(GR-ENG-1, GR-ENG-2 and GR-ENG-4), *Heterodera glycines *(HG-ENG-2, HG-ENG-4, HG-ENG-5 and HG-ENG-6), *Ditylenchus africanus *(DA-ENG-1), beetles, bacteria and protists are indicated in brackets. The bootstrap values are calculated from 1000 replicates. The scale bar represents 10 substitutions per 100 amino acid positions.

A phylogenetic tree was generated from an alignment of the β-1,4-endoglucanase protein sequences from AA-ENG-1, AA-ENG-2, cyst and root-knot nematodes, the migratory plant-parasitic nematodes *R. similis, Pratylenchus penetrans, Pratylenchus coffeae *and *Ditylenchus africanus *and GHF5 cellulases from phytophagous beetles, bacteria and protists (Fig. 6). AA-ENG-1 and AA-ENG-2 clustered into a larger group of protein sequences including all nematode GHF5 cellulases, indicating that *A. avenae *cellulases are closely related to those of the Tylenchida. This analysis supports the idea that all nematode GHF5 cellulases evolved from a GHF5 sequence acquired by a common ancestor of this group.

The β-1,4-endoglucanases are the largest family of cell-wall-degrading enzymes that have been identified in parasitic nematodes to date. Over the last decade, a large number of GHF5 endoglucanases have been identified and extensively studied in plant parasitic Tylenchida including cyst and root-knot nematodes [20-25]. Genes encoding β-1,4-endoglucanases have also been found in *Bursaphelenchus *spp but these enzymes are most similar to GHF45 cellulases from fungi [16]. The presence of GHF5 cellulases within *A. avenae *(as opposed to GHF45 cellulases) provides further support for the suggestion that this nematode is more closely related to the Tylenchida than to *Bursaphelenchus *and its relatives.

Although the presence of GHF5 cellulases within *A. avenae *can be readily explained given the phylogenetic arguments above, the presence of a β-1,3-endoglucanase is more surprising. These enzymes act to metabolise the fungal cell wall and have been previously described in *Bursaphelenchus *spp. Such genes are not usually present in animals and it was suggested that the *Bursaphelenchus *genes were acquired by horizontal gene transfer from bacteria [17].

No such genes are present in root-knot nematodes (for which two genome sequences are available) or other Tylenchida. It is possible that a fungal feeding common ancestor of Aphelenchoidea and Tylenchida possessed this gene but that more "advanced" plant parasites have subsequently lost it. Further sequencing within both nematode groups is required to resolve this issue.

All the transcripts potentially encoding pectate lyases were identified as singletons (Table 4). Two full-length cDNA sequences of pectate lyases, designated *Aa-pel-1 *and *Aa-pel-2*, were identified from the plasmid clones corresponding to the cluster IDs AAC01649 and AAC03048 respectively. The complete cDNA of *Aa-pel-1 *was 821 bp in length and contained an ORF of 247 amino acids with a putative ATG start codon at position 30 and TAA stop codon at position 771. A signal peptide of 18 amino acids is predicted by SignalP [63] at the N-terminus of the putative AA-PEL-1 amino acid sequence. The mature protein has a predicted molecular mass of 24.250 kDa and theoretical pI of 8.93.

The full-length *Aa-pel-2 *cDNA was 838 bp long and contained an ORF of 249 bp with an ATG start codon at position 31 and a TAA stop codon at position 778. An N-terminal signal sequence of 19 amino acids predicted by SignalP [63]. The molecular mass and theoretical pI value of the putative AA-PEL-2 protein were 24.329 kDa and 9.12 respectively. AA-PEL-1 has 61% identity to AA-PEL-2.

**Figure 7:**
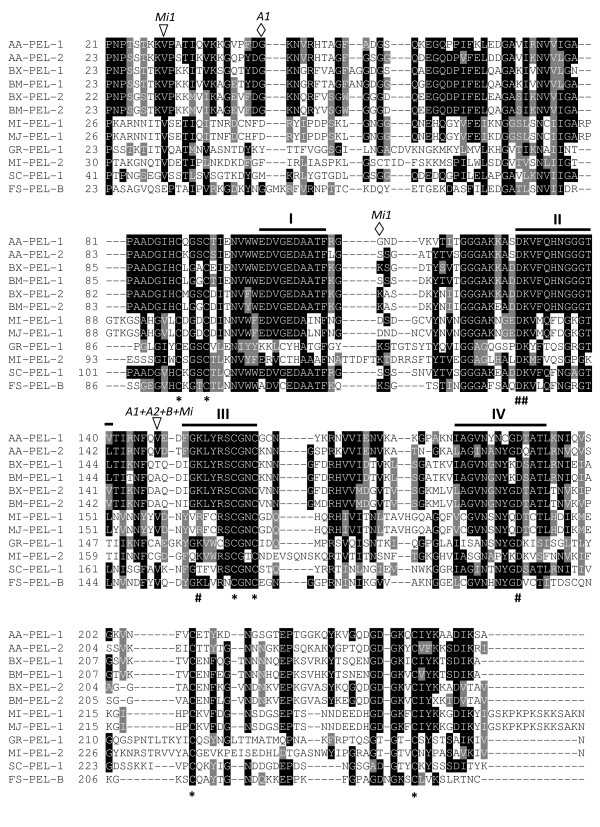
**Multiple sequence alignment of *A. avenae *pectate lyase protein sequences (AA-PEL-1 and AA-PEL-2) with the sequences of pectate lyases from plant parasitic nematodes, bacterium, and a fungus**. BX-PEL-1 [BAE48369], BX-PEL-2 [BAE48370] from *Bursaphelenchus xylophilus*; BM-PEL-1 [BAE48373], and BM-PEL-2 [BAE48375] from *Bursaphelenchus mucronatus*; MI-PEL-1 [AAQ09004] and MI-PEL-2 [AAQ97032] from *Meloidogyne incognita*; MJ-PEL-1 [AAL66022] from *Meloidogyne javanica*; GR-PEL-1 [AAF80747] from *Globodera rostochiensis*; SC-PEL-1 [NP625403] from *Streptomyces coelicolor *and FS-PEL-B [AAA8738] from *Fusarium solani*. Identical residues are highlighted in black and functionally conserved are in gray. Black bars (I to IV) indicate the conserved regions characteristic of PL3 pectate lyases. Very highly conserved charged residues are indicated beneath the alignment by a number symbol (#), while an asterisk (*) indicates conserved cysteine residues. The positions of the intron in AA-PEL-1 and AA-PEL-2 are indicated by *A1 *and *A2 *respectively, that in BX-PEL-1/2 and BM-PEL-1/2 by *B*, and those in MI-ENG-1 by *Mi1*. Triangles and diamonds represent phase 0 and 1 introns, respectively.

To obtain genomic sequences, the entire coding regions of *Aa-pel-1 *and *Aa-pel-2 *gene were amplified from *A. avenae *gDNA using gene specific primers (Table 5). Analysis of these sequences showed that the *Aa-pel-1 *and *Aa-pel-2 *genes were 1,860 and 1,221 bp long respectively from the ATG to the stop codon. Two introns (468 bp and 651 bp) were identified in *Aa-pel-1 *whereas *Aa-pel-2 *contained only one intron (690 bp) (Fig. 7). All introns were bordered by canonical cis-splicing sequences [65]. The position of the second intron of *Aa-pel-1 *was identical to the intron in *Aa-pel-2*.

The intron position in the *A. avenae *genes (*Aa-pel-1 *and *Aa-pel-2*) were compared with other nematode pectate lyase genes. The pectate lyase genes from *Bursaphelenchus *species (*Bx-pel-1/2 and Bm-pel-1/2*) each have one intron in their coding region at the same position [18]. This position is also identical to the common intron position of the *A. avenae *genes (Fig. 7). Moreover, one of the three introns of *Mi-pel-1 *from *M. incognita *is at the same position as that in the *A. avenae *and *Bursaphelenchus *genes. *Gr-pel-1 *from *G. rostochiensis *has six introns and *Mi-pel-2 *from *M. incognita *has two introns. *Gr-pel-1 *and *Mi-pel-2 *share two intron positions but none of the introns of these genes have the same position as that in the *A. avenae *genes (not shown).

A protein homology search using the deduced amino acid sequences of AA-PEL-1 and AA-PEL-2 using BLASTP indicated high similarity to the pectate lyases belonging to the the polysaccharide lyase family 3 (PL3) from plant parasitic nematodes, bacteria and fungi. Multiple sequence alignment of AA-PEL-1 and AA-PEL-2 with the best matches confirmed that both *A. avenae *sequences contained the four highly conserved regions characteristic of PL3 pectate lyases in bacteria and fungi as well as 8-10 cysteine residues and four charged residues (Fig. 7) that are potentially involved in catalysis [26,68]. AA-PEL-1 and AA-PEL-2 were most similar to the pectate lyases (BX-PEL1/2 and BM-PEL1/2) from the pinewood nematodes *B. xylophilus *and *B. mucronatus *(52 to 59% identity for the AA-PEL-1 and 53 to 63% identity for AA-PEL-2) [18]. *A. avenae *sequences shared 23 to 33% identity with pectate lyases (MI-PEL-1, MI-PEL-2, MJ-PEL-1 and GR-PEL-1) from cyst and root-knot plant parasitic nematode spp [28,29], and 39 to 45% identity with the sequences from two microbes (Fig. 7).

**Figure 8:**
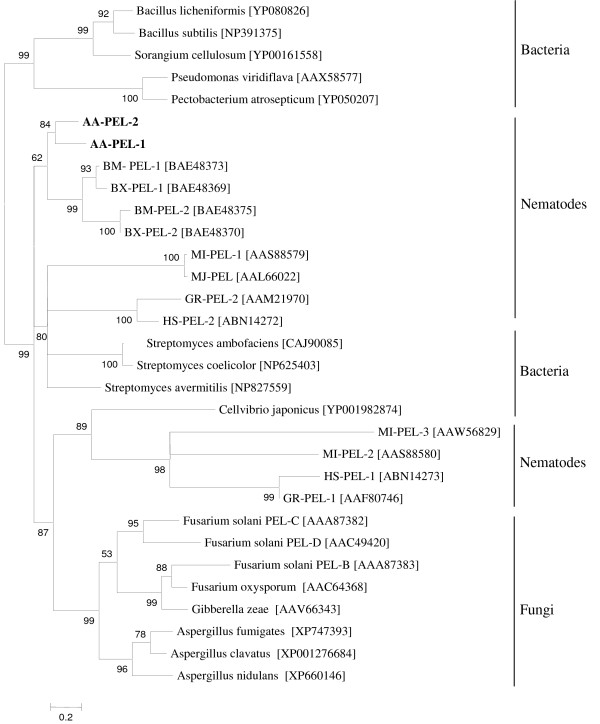
**Unrooted phylogenetic tree of selected polysaccharide lyase family 3 proteins generated using maximum likelihood analysis**. The PL3 proteins from *A. avenae *(AA-PEL-1 and AA-PEL-2) are labeled in bold. GenBank accession numbers of PL3 proteins from *Bursaphelenchus mucronatus *(BM-PEL-1 and BM-PEL-2), *Bursaphelenchus xylophilus *(BX-PEL-1 and BX-PEL-2), *Meloidogyne incognita *(MI-PEL-1, MI-PEL-2 and MI-PEL-3), *Meloidogyne javanica *(MJ-PEL), *Globodera rostochiensis *(GR-PEL-1 and GR-PEL-2), *Heterodera schachtii *(HS-PEL-1 and HS-PEL-2), bacteria and fungi are indicated in brackets. The bootstrap values are calculated from 1000 replicates. The scale bar represents 20 substitutions per 100 amino acid positions.

A phylogenetic tree was generated from an alignment of *A. avenae *pectate lyase sequences with selected proteins belonging to PL3 from bacteria, fungi, and nematodes using the maximum likelihood method (Fig. 8). Both *Aa-pel-1 *and *Aa-pel-2 *were clustered with the *Bursaphelenchus genes*. Other nematode sequences were not monophyletic but were clustered into distinct clades.

**Figure 9:**
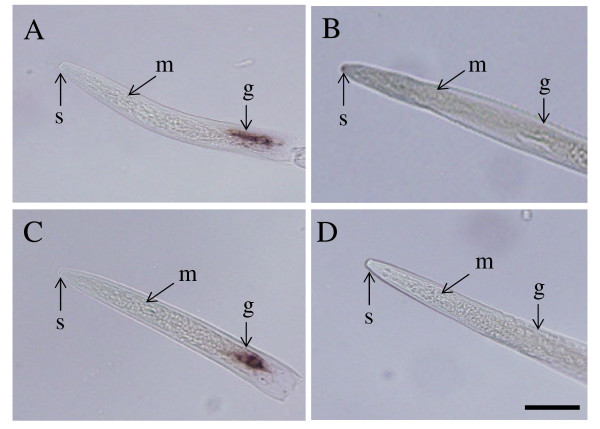
**Localization of *Aa-eng-1 *and *Aa-pel-1 *transcripts in the esophageal gland cells of *A. avenae *by *in situ *hybridisation**. Nematode sections were hybridized with antisense (A) and sense (B) *Aa-eng-1 *digoxigenin-labeled cDNA probes. Hybridisation was also carried out with digoxigenin-labeled antisense (C) and sense (D) *Aa-pel-1 *cDNA probes. G, esophageal glands; S, stylet; M, metacorpus. The bar indicates a length of 20 μm.

The pectate lyase genes from *A. avenae *are more similar to the *Bursaphelenchus *genes compared to those from cyst and root-knot nematodes (Figs. 7 and 8). The identical position of the common intron of the *A. avenae *genes (*Aa-pel-1 *and *Aa-pel-2*) and introns within pectate lyase genes from *Bursaphelenchus *and *M. incognita *(Fig. 7) suggests that pectate lyase genes from a wide range of plant parasitic nematodes have the same origin. To determine which *A. avenae *cells express GHF5 endoglucanases and pectate lyases, *in situ *mRNA hybridisation was performed (Fig. 9). Digoxigenin-labeled antisense probes generated from *Aa-eng-1 *and *Aa-pel-1 *specifically hybridized with the transcripts in the esophageal gland cells of *A. avenae *(Figs. 9A and 9C). Staining was observed in juvenile and adult nematodes rather than being restricted to a specific life stages. No hybridisation was observed with the control (sense) cDNA probes of *Aa-eng-1 *or *Aa-pel-1 *(Figs. 9B and 9D).

As a part of the complex process of parasitism, a wide range of plant parasitic nematodes use endogenous β-1,4-endoglucanases and pectate lyases to degrade two abundant constituents of the plant cell wall and thus facilitate their migration through host tissues. The presence of signal peptides in the deduced amino acid sequences of the endoglucanases and pectate lyase from *A. avenae *coupled with their expression in the esophageal glands suggest that both enzymes have a similar role in *A. avenae*. The presence of such genes in *A. avenae *suggests that this nematode can enter and migrate through plant tissues and may also be able to feed on plant cell contents. This is backed up by the observation that *A. avenae *is known to feed on plant tissue in culture [13,14]. *A. avenae *may therefore have a wide ranging diet that includes fungi and plant tissues. This, coupled with the position of this nematode as a basal member of a clade that includes a wide range of plant parasitic nematodes, provides further support for the idea that plant parasitism has evolved from fungal feeding and suggests that *A. avenae*, may be a very primitive plant feeder.

## Conclusion

The 5,076 ESTs identified in this study represent the first attempt to define the *A. avenae *gene set and represents over 2,200 genes. This collection of ESTs represents a starting point for studies in a number of different fundamental and applied areas. A summary of the assignment of nonredundant ESTs to functional categories as well as their relative abundance are listed and discussed. A substantial number of putative *Aphelenchus*-specific genes were found that do not share similarity with known genes and some of these may be highly expressed, based on their abundance in the EST dataset. The presence of genes encoding a battery of cell-wall-degrading enzymes in *A. avenae *and their similarities with the genes from other plant parasitic nematodes suggest that this nematode can act not only as a fungal feeder but also as a plant parasite. The gene structures of GHF5 cellulase and PL3 pectate lyase from *A. avenae*, their phylogenetics and comparative analyses with similar genes from other parasitic nematodes provides information that helps understand the evolutionary origins of these genes within the Nematoda. Further studies on genes encoding cell-wall-degrading enzymes in *A. avenae *will accelerate our understanding of the complex evolutionary histories of plant parasitism and the use of genes obtained by horizontal gene transfer from prokaryotes.

## Methods

### Biological material

The AaF1 isolate of *A. avenae *was cultured on fungi, *Botrytis cinerea *for 2-3 weeks at 25°C and then extracted for 3 h at 25°C using the modified Baermann funnel technique [69]. Separated, mixed life stage, nematodes were cleaned by flotation on a 30% (wt/vol) sucrose solution followed by three washes with 0.5× PBST [70]. Nematodes were stored at -80°C until use.

### Isolation of total RNA, cDNA synthesis and cDNA library construction

Total RNA from mixed stage *A. avenae *was isolated using Sepasol (Nakalai). Analysis of the total RNA on a denaturing agarose gel showed a smear from 50 to 3,000 bp with two distinct bands of ribosomal RNA. Poly(A)+ RNA was extracted from total RNA using a FastTrack^® ^MAG micro mRNA Isolation Kit (Invitrogen). cDNA was synthesized using the SMART PCR cDNA amplification method (Clontech) with a *Not*I oligo-dT primer (5' AACTGGAAGAATTCGCGGCCGCAGGAATTTTTTTTTTTTTTTTTT). *SalI/Sma*I adaptors (Takara) were added to double stranded cDNA which was digested with *Not*I and size fractionated using a cDNA Size Fractionation Column to remove small cDNA (< 500 bp) (Invitrogen). The appropriate fractions containing cDNA were pooled and ethanol precipitated. Inserts were directionally cloned into *Not*I and *Sal*I sites of the pSPORT1 vector, and transformed into *Escherichia coli *DH5α cells. The cDNA library was designated as Aamk.

### EST generation

Individual transformants (n = 5,472) from the plasmid library were picked into 96 well plates containing 0.5 ml of LB medium containing 100 μg/ml ampicillin. Plates were incubated overnight at 37°C. A small aliquot of each culture was stored at -80°C after being mixed with same volume of 25% glycerol in LB. Plasmid DNA was isolated and purified using FB glass fiber plates (Millipore) using the glass bead method described in [71]. cDNA inserts were sequenced from the 5' end using the M13-T7 primer (5'-TAATACGACTCACTATAGGG-3') and the BigDye terminator ver. 3.1 kit (Applied Biosystems) on an ABI 3100 DNA sequencer (Applied Biosystems). Raw sequence trace data from the 3100 sequencer were processed in an automated pipeline, the trace2dbEST package [72]. Before submitting them to the public database (DDBJ), sequences were processed to assess quality, remove vector sequence, contaminants and cloning artifacts and to identify BLAST similarities.

### Clustering and sequence analysis

Clustering was performed using PartiGene, a software pipeline designed to analyze and organize EST data sets [72]. Sequences were clustered into groups (putative genes) on the basis of sequence similarity using CLOBB [73]. Clusters were assembled to yield consensus sequences using Phrap (P. Green, unpublished data). Each consensus sequence was subjected to BLAST analysis against the GenBank non-redundant protein database.

Comparative analyses were performed with cluster sequences as queries versus multiple databases including *C. elegans *Wormpep v.203 protein database [46]. Protein databases for 'other nematode' and 'non-nematodes' were generated in-house for similarity searches. The 'other nematode'database contained all available protein sequences from nematodes other than *C. elegans *and *A. avenae *as well as nematode ESTs in GenBank (April 30, 2009), translated into peptide sequences (in TBLASTX analysis). The 'non-nematode' database comprises amino acid sequences from the complete non-redundant protein database (April 30, 2009) excluding those from nematodes. Homologues to cluster sequences were identified *via *comparisons against WormBase and non-redundant protein databases using the BLASTX algorithm. The TBLASTX algorithm with default parameters was used to compare the *A. avenae *cluster sequences to ESTs from other nematodes. Each cluster sequence of *A. avenae *was assigned a 'statistically significant' match if the E-value from the BLAST output of the sequence alignment was < 1e-05. The program SimiTri [48] was used for the comparison (at the amino acid sequence level) of *A. avenae *cluster sequences with data in *C. elegans*, other nematode and non-nematode protein sequence databases, providing a two dimensional display of relative similarity relationships among the three different datasets. "Fragmentation" defined as the representation of a single gene by multiple non-overlapping clusters, was estimated by comparing *A. avenae *cluster sequences with *C. elegans *[39].

### Gene ontology (GO)

Cluster sequences were classified into Gene Ontology functional categories [54] based on BLAST similarities to known genes in the NCBI Swiss-Prot protein sequence (Swiss-Prot) and using the BLAST2GO annotation tool [55,56] with an E-value cut-off of 1e-05 and summarized according to their biological processes, molecular functions and cellular components. To obtain the complete GO mapping, a node sequence filter in the GO graph was used 50 for biological process, 20 for molecular function, and 20 for the cellular component. The multi-level pie charts were generated using sequence cut-offs of 140, 50 and 40 for biological process, molecular function and cellular components respectively.

### Identification of genes encoding cell-wall-degrading enzymes and sequence analyses

BLASTX searches against the GenBank database were used to identify *A. avenae *clusters encoding potential cell-wall-degrading enzymes. To obtain full length sequences of genes, the plasmid clones from which each of these sequences were obtained were identified and re-sequenced both directions using designed primers (Table 5) in order to obtain the full-length cDNA sequences. The genomic coding region of each cDNA clone was obtained by PCR amplification from *A. avenae *genomic DNA, using pairs of gene-specific primers flanking each open reading frame. PCR products were cloned into the pGEM-T Easy vector (Promega) and sequenced using standard protocols.

Signal peptide predictions were made using the SignalP program [63]. Protein theoretical pI and molecular mass were predicted using the "Compute pI/M_W _tool" available at ExPASy http://br.expasy.org/tools/pi_tool.html. Sequence alignments were made with a multiple alignment program, MAFFT version 6 http://align.bmr.kyushu-u.ac.jp/mafft/software/about.html and output was produced using BOXSHADE 3.21 http://align.bmr.kyushu-u.ac.jp/mafft/software/about.html

### Phylogenetic analyses

The phylogenetic analyses of the catalytic domains of the GHF5 proteins and PL3 proteins were performed on the Phylogeny.fr platform [74] and comprised the following steps. Sequences were aligned with MAFFT (v. 6) configured for highest accuracy (MAFFT with E-INS-i option). Prior to the phylogenetic analysis, signal peptide sequences and other N and C terminal extentions peculiar to individual taxa were excluded. In total 337 and 249 characters were used for GHF5 endoglucanase and pectate lyase respectively for phylogenetic anlaysis. The phylogenetic tree was reconstructed using the maximum likelihood method implemented in the PhyML program (v3.0 aLRT). The JTT substitution model was selected assuming an estimated proportion of invariant sites (of 0.018) and 4 gamma-distributed rate categories to account for rate heterogeneity across sites. The gamma shape parameter was estimated directly from the data (gamma = 1.603). Reliability for internal branch was assessed using the aLRT test (SH-Like). Graphical representation and edition of the phylogenetic tree were performed with TreeDyn (v198.3).

### *In situ *hybridisation

*In situ *hybridisation was performed as described previously[75]. PCR products were generated from the plasmid stocks of the cDNA clones of *Aa-eng-1 *and *Aa-pel-1 *using gene specific primers (Table 5). Sense or antisense stra nds were labelled with digoxigenin by asymmetric PCR and hybridised to fixed, permeabilised fragments of mixed stage *A. avenae*. After washing to remove unbound probe, specifically hybridising probe was detected using Alkaline-phosphatase conjugated Anti-Digoxigenin antibody and NBT/BCIP stock solution (Roche Diagnostics). Specimens were examined with differential interference microscopy (Nikon).

### Sequence Data

All EST sequences described in this article have been deposited in the dbEST division of GenBank under the accession numbers [GenBank:GO 479265] - [GenBank: GO484340]. The mRNA and gDNA sequences of GHF5 and PL3 have been deposited in the DDBJ under the accession numbers [DDBJ: AB495300 (*Aa-eng-1 *mRNA)], [DDBJ:AB495301 (*Aa-eng-1 *gDNA)], [DDBJ:AB495302 (*Aa-eng-2 *mRNA)], [DDBJ:AB495303 (*Aa-eng-2 *gDNA)], [DDBJ:AB495304 (*Aa-pel-1 *mRNA)], [DDBJ:AB495305 (*Aa-pel-1 *gDNA)], [DDBJ:AB495306 (*Aa-Pel-2 *mRNA)] and [DDBJ:AB495307 (*Aa-pel-2 *gDNA).

## Authors' contributions

NK and TK conceived and designed the research plan and executed all of the experiments. HO provided the *A. avenae *isolate used in this study. HO and JTJ coordinated the project. NK wrote the manuscript. TK contributed to the bioinformatics analysis and assisted in preparing the manuscript. JTJ interpreted results, provided valuable assistance in preparing the manuscript. All authors contributed to, read and approved the final manuscript.

## Supplementary Material

Additional file 1 **Venn diagram illustrating the distribution of BLAST hits for "other nematode" (other than *C. elegans*) specific *A. avenae *cluster sequences**. Positive hits were identified for the BLASTX and TBLASTX searches of 137 sequences (Fig. 3) and matches were found in "plant parasitic", "animal parasitic and other", and "free living" nematodes. Thirty four *A. avenae *cluster sequences produced significant match (E < 1e-5) only to sequences from plant parasitic nematodes. Click here for file

Additional file 2 ***A. avenae *transcripts similar to stress-response genes related to desiccation**. BLASTX searches (E < 1e-5) of 2,700 cluster sequences against non redundant protein databases allowed identification of some genes that can encode proteins or enzymes known to be associated with desiccation-stress of nematodes. Click here for file

Additional file 3 **Gene Ontology mappings (using GO slim terms) for *A. avenae *clusters. Note that individual GO categories can have multiple mappings**. To obtain the complete GO mapping, a node sequence filter in the GO graph was used 50 for biological process, 20 for molecular function, and 20 for the cellular component. Click here for file
