# Supplementary material for: Analysis of expressed sequence tags and identification of genes encoding cell-wall-degrading enzymes from the fungivorous nematode Aphelenchus avenae
Source: BMC Genomics. 2009 Nov 16;10:525. doi: 10.1186/1471-2164-10-525 (PMC2784482; doi:10.1186/1471-2164-10-525)
Supplement: Additional file 1 — Venn diagram illustrating the distribution of BLAST hits for "other nematode" (other than C. elegans) specific A. avenae cluster sequences. Positive hits were identified for the BLASTX and TBLASTX searches of 137 sequences (Fig. 3) and matches were found in "plant parasitic", "animal parasitic and other", and "free living" nematodes. Thirty four A. avenae cluster sequences produced significant match (E < 1e-5) only to sequences from plant parasitic nematodes. [file 1471-2164-10-525-S1.DOC]

**Additional file 1.** **Venn diagram illustrating the distribution of BLAST hits for “other nematode” (other than *C. elegans*) specific *A. avenae* cluster sequences.**
