# Supplementary material for: Analysis of expressed sequence tags and identification of genes encoding cell-wall-degrading enzymes from the fungivorous nematode Aphelenchus avenae
Source: BMC Genomics. 2009 Nov 16;10:525. doi: 10.1186/1471-2164-10-525 (PMC2784482; doi:10.1186/1471-2164-10-525)
Supplement: Additional file 2 — A. avenae transcripts similar to stress-response genes related to desiccation. BLASTX searches (E < 1e-5) of 2,700 cluster sequences against non redundant protein databases allowed identification of some genes that can encode proteins or enzymes known to be associated with desiccation-stress of nematodes. [file 1471-2164-10-525-S2.DOC]

**Additional file 2.** *A. avenae* transcripts similar to stress-response genes related to desiccation

| Protein  name | Cluster ID | ESTs | Non-redundant Genebank | | | | |
| --- | --- | --- | --- | --- | --- | --- | --- |
|  |  |  | Best identity descriptor | Accession | | E-value | |
|  |  | | | | | | |
| LEA protein | AAC00729 | 7 | LEA5 protein [*Steinernema carpocapsae*] | | ABQ23240 | | 7e-12 |
|  | AAC00888 | 5 | Group 3 late embryogenesis abundant protein [*Phaseolus vulgaris*] | | ABA26579 | | 1e-28 |
|  | AAC01781 | 1 | Late embryogenesis abundant protein 1 (Aavlea1) [*A. avenae*] | | Q95V77 | | 3e-17 |
|  |  |  |  | |  | |  |
|  |  |  |  | |  | |  |
| Cytochrome P450 | AAC00691 | 1 | Cytochrome P450 family member (cyp-33C9) [*C. elegans*] | | NP_503846 | | 2e-25 |
|  | AAC00853 | 1 | Cytochrome P450 family member (cyp-14A3) [*C. elegans*] | | NP_510205 | | 1e-38 |
|  | AAC01309 | 1 | Cytochrome P450 family member (cyp-33E1) [*C. elegans*] | | NP_501480 | | 1e-20 |
|  | AAC01377 | 1 | Hypothetical protein CBG21823 [*C. briggsae* ] | | XP_001672714 | | 6e-12 |
|  | AAC01499 | 1 | Cytochrome P450 family member (cyp-33C2) [*C. elegans*] | | NP_503594 | | 3e-54 |
|  | AAC01503 | 1 | Cytochrome P450 family member (cyp-14A3) [*C. elegans*] | | NP_510205 | | 7e-43 |
|  | AAC01643 | 1 | Hypothetical protein TRIADDRAFT_25960 [*Trichoplax adhaerens*] | | XP_002112322 | | 9e-15 |
|  | AAC02063 | 1 | Cytochrome P450 family member (cyp-33D3) [*C. elegans*] | | NP_507679 | | 2e-22 |
|  | AAC02116 | 1 | Cytochrome P450 family member (cyp-33D1) [*C. elegans*] | | NP_507076 | | 7e-27 |
|  | AAC02145 | 1 | Cytochrome P450 family member (cyp-14A2) [*C. elegans*] | | NP_510204 | | 9e-32 |
|  | AAC02155 | 1 | Cytochrome P450 family member (cyp-33E1) [*C. elegans*] | | NP_501480 | | 3e-19 |
|  | AAC02503 | 1 | Cytochrome P450 family member (cyp-33C9) [*C. elegans*] | | NP_503846 | | 3e-37 |
|  | AAC02643 | 1 | Cytochrome P450 family member (cyp-33A1) [*C. elegans*] | | NP_504988 | | 5e-17 |
|  | AAC02834 | 1 | Cytochrome P450 family member (cyp-33C9) [*C. elegans*] | | NP_503846 | | 6e-31 |
|  |  |  |  | |  | |  |
|  |  |  |  | |  | |  |
| Superoxide | AAC01835 | 1 | Superoxide dismutase [*Aedes aegypti*] | | XP_001654772 | | 6e-29 |
| dismutase | AAC03099 | 1 | Superoxide dismutase [*Haemonchus contortus*] | | Q27666 | | 6e-39 |
|  |  |  |  | |  | |  |
|  |  |  |  | |  | |  |
| Glutathion | AAC00445 | 5 | Glutathione peroxidase [*H. contortus*] | | AAT28332 | | 2e-101 |
| peroxidase | AAC00790 | 1 | Glutathione peroxidase [*Azolla pinnata*] | | ABQ96603 | | 2e-09 |
|  | AAC02098 |  | Putative glutathione peroxidase [*Glossina morsitans morsitans*] | | AAT85827 | | 3e-16 |
|  | AAC02765 | 1 | Phospholipid-hydroperoxide glutathione peroxidase [*Dermacentor variabilis*] | | ACF35507 | | 3e-21 |
|  | AAC03080 | 1 | Secreted glutathione peroxidase [*G. rostochiensis*] | | CAD38523 | | 3e-35 |
|  |  |  |  | |  | |  |
|  |  |  |  | |  | |  |
| Glutathion | AAC00194 | 1 | Glutathione S-Transferase family member (gst-30) [*C. elegans*] | | NP_494902 | | 8e-29 |
| S-transferase | AAC00758 | 2 | Glutathione S-Transferase family member (gst-7) [*C. elegans*] | | NP_494883 | | 4e-12 |
|  | AAC00832 | 1 | Glutathione S-transferase [*H. contortus*] | | AAF81283 | | 8e-21 |
|  | AAC00961 | 4 | Glutathione S-Transferase family member (gst-36) [*C. elegans*] | | NP_509652 | | 5e-11 |
|  |  |  |  | |  | |  |
